# Comparison of postoperative liver function between different dissection techniques during laparoscopic cholecystectomy

**DOI:** 10.2144/fsoa-2019-0160

**Published:** 2020-02-07

**Authors:** Tagleb S Mazahreh, Abdelwahab J Aleshawi, Nabil A Al-Zoubi, Mohammad Altabari, Qusai Aljarrah

**Affiliations:** 1 Department of General Surgery & Urology, Faculty of Medicine, Jordan University of Science & Technology, Irbid 22110, Jordan; 2 King Abdullah University Hospital, Jordan University of Science & Technology, Irbid 22110, Jordan

**Keywords:** bilirubin, Hook dissector, laparoscopic cholecystectomy, liver function tests, Maryland dissector

## Abstract

**Aim:**

In this study, we investigated and compared the effect of different types of dissector (Maryland vs Hook) on changes in liver function tests (LFTs) after laparoscopic cholecystectomy.

**Patients & methods:**

The enrolled patients were divided into two groups. Group A patients underwent dissection by Maryland dissecting forceps, group B by Hook dissecting instrument. LFTs were measured preoperatively and at 1 day and 1 week, postoperatively.

**Results:**

For both Maryland and Hook dissection, the 1-day postoperative values for total bilirubin, alanine aminotransferase and aspartate aminotransferase were significantly higher than the preoperative values. Also, there were no statistical differences between Hook and Maryland.

**Conclusion:**

The elevation of LFTs seems to be attributed to other factors.

## Lay abstract

Laparoscopic cholecystectomy (LC) is an alternative to laparotomy and has become the standard treatment of benign gallbladder diseases. However, it has been noted that (following LC) the serum level of certain liver function tests (LFT) raises markedly in patients who had preoperatively normal LFT. Pneumoperitoneum is the main contributing factor. This is the first study to evaluate the effect of different dissectors on alteration of LFTs after LC. As there were no statistical differences in the variation of LFTs between the Maryland and Hook, it seems that the dissector type has no effect on the alteration of LFTs.

Laparoscopic cholecystectomy (LC) is the gold standard treatment for managing symptomatic cholelithiasis. Its advantages include reduced patient discomfort, better cosmetic results, shorter hospital stay and shorter interval to return to work [1,2]. However, LC can impair liver function tests (LFTs). Elevation in LFTs following LC is an apparent cause of apprehension to the surgeon concerned about the integrity of the biliary tree [3–10].

The observation of postoperative changes in the level of LFTs after LC was first reported in the literature by Halevy *et al.* who demonstrated an increase of up to 80% from the baseline level of LFTs, without adverse clinical outcome [11]. It was assumed that CO2 pneumoperitoneum and increased intra-abdominal pressure are the main reasons for these changes [7,10,12]. Also, the disturbance in LFTs can be caused by the hepatotoxic effect of anesthetic drugs (halothane, nitrous oxide) [2,13]. However, to the best of our knowledge, no study was conducted to evaluate the effect of different types of dissectors used in LC on the alteration of LFTs. Therefore, we have conducted this study to investigate and compare the effect of different types of dissector (Maryland vs Hook) on changes in LFTs.

## Patients & methods

This study was conducted at King Abdullah University Hospital, a tertiary care center that is affiliated with the Jordan University of Science and Technology, located in northern Jordan. After obtaining the Institutional Review Board approval, we prospectively selected patients who underwent LC between June 2017 and December 2018. The following data were obtained: demographics (age and sex), indication for surgery, the surgical instrument used to dissect gallbladder bed, preoperative LFTs and postoperative LFTs at first day and 1 week postoperatively.

Patients admitted as a case of acute cholecystitis, ascending cholangitis, gallbladder stones with obstructive jaundice, gallbladder stones with pancreatitis, history of chronic liver disease, gallbladder empyema, gangrene or perforation and gallbladder malignancy were excluded. Moreover, patients who experienced postoperative biliary tree complications were also excluded. Only patients who were admitted electively for gallbladder stones were included in the study. The LFTs that were ordered pre and postoperatively were: ALP, ALT, AST, GGT, direct bilirubin and total bilirubin.

Patients were allocated randomly into two groups. Group A included patients who underwent dissection of the gallbladder by the Maryland dissecting forceps connected to the electro-cautery device, while group B included patients who underwent dissection of the gallbladder by Hook instrument, connected to the same electro-cautery device.

### Setting

LC operations were performed by a single surgeon and following the standard method. In all patients, the same electrocautery device (Karl–Storz) was utilized on a monopolar electrode and coagulation waveform mode, with a pre-adjusted power of 30 w. Group A underwent standard LC using Maryland dissecting forceps (Karl–Storz 36 cm length). Group B underwent standard LC using Hook dissecting instrument (Karl–Storz 36 cm length).

Patients brought to the operating room received a shot of 1 g iv. cefazolin preoperatively, after the formal surgical safety checklist was performed. The abdomen was prepared and draped in a sterile fashion, in a supine position, after general anesthesia and intubation. Supra-umbilical midline incision was made and open technique was used to enter the peritoneal cavity and to establish the pneumoperitoneum at a standard pressure of 14 mmhg. The peritoneal cavity was inspected without abnormalities in all patients.

The patient was placed in reverse Trendelenburg position with the right side up. Omental attachments to the gallbladder were gently swept away until an atraumatic grasper could be used to retract the fundus of the gallbladder, superior-laterally over the dome of the liver. The infundibulum was identified and subsequently retracted laterally, toward the right lower quadrant using another grasper. This maneuver exposed Calot's triangle. The peritoneum overlying the gallbladder infundibulum was incised with either Maryland in group A or Hook in group B, anteriorly. The triangle was dissected to expose the cystic duct, the cystic artery and lymph node. Once these structures were carefully identified and the critical view of safety was adequately achieved, the cystic artery was divided first, followed by further dissection of the triangle until it was determined that the only structure remaining entering the gallbladder was the cystic duct, it was then doubly clipped and divided.

Next, the peritoneal attachments between the gallbladder and its liver bed were dissected (by Maryland in group A and by Hook in group B). The gallbladder fossa and cystic artery stump were inspected to ensure adequately secured hemostasis. The gallbladder, once freed, was placed in an endoscopic retrieval bag and removed from the abdomen through the supra-umbilical port. The same anesthetic protocol was used for both group of patients. The Hook and Maryland dissectors are of the reusable types. After each operation, both dissectors undergo the same sterilization procedure. Accordingly, the total cost for each dissector is the same.

### Statistical analysis

Statistical analyses were performed using IBM SPSS Statistics Software (v.21), 2012. Categorical variables were described using the frequency distribution, while continuous variables were described using the mean ± standard error of the mean. Data were blocked into two groups and examined at the 95% confidence interval using Pearson's chi-square test of association for categorical variables and student's *t*-test for continuous variables, after testing for distribution normality.

## Results

A total of 137 patients who underwent elective LC for gallstones were included in the study; 86 patients were excluded. The analyzed patients were divided into two groups; A and B. Group A included 68 patients (28 males and 40 females), while group B was composed of 69 patients (24 males and 45 females). The mean age for group A and B was 46.5 and 40.0 (p > 0.05), respectively.

The patients mean preoperative and postoperative LFTs values are illustrated in Table 1. For the Maryland, there was no statistical difference between the preoperative and 1-day postoperative values for ALP, direct bilirubin and GGT. However, the 1-day postoperative values for total bilirubin, ALT and AST were significantly higher than the preoperative values. Similarly, for the Hook in group B, there was no statistical difference between the preoperative and 1-day postoperative values for ALP, direct bilirubin and GGT. However, the values for total bilirubin, ALT and AST were significantly elevated 1-day postoperatively. The mean duration for gallbladder bed dissection was not statistically different in both groups (13.8 min for Maryland and 14.2 min for the Hook).

**Table 1.** Preoperative and 1-day postoperative measurement of liver function tests for each dissector.

Procedure type	Liver function test	Mean ± SE	Changes in mean	p-value
Group 1, Maryland dissector (N = 68)	Preoperative ALP	169.70 ± 11.15		
	Postoperative ALP	161.15 ± 9.35	8.55	NS
	Preoperative total bilirubin	10.69 ± 0.86		
	Postoperative total bilirubin	13.55 ± 1.49	-2.87	0.006
	Preoperative direct bilirubin	4.74 ± 0.59		
	Postoperative direct bilirubin	5.87 ± 0.92	-1.13	NS
	Preoperative ALT	36.07 ± 4.83		
	Postoperative ALT	61.09 ± 8.96	-25.02	0.005
	Preoperative AST	26.43 ± 2.46		
	Postoperative AST	49.30 ± 6.59	-22.87	0.001
	Preoperative GGT	71.75 ± 13.60		
	Postoperative GGT	77.79 ± 11.03	-6.04	NS
Group 2, Hook dissector (N = 69)	Preoperative ALP	156.88 ± 9.64		
	Postoperative ALP	155.93 ± 9.51	0.95	NS
	Preoperative total bilirubin	9.42 ± 0.74		
	Postoperative total bilirubin	12.67 ± 1.42	-3.25	0.002
	Preoperative direct bilirubin	3.53 ± 0.57023		
	Postoperative direct bilirubin	4.67 ± 1.04	-1.14	NS
	Preoperative ALT	31.20 ± 3.53		
	Postoperative ALT	48.34 ± 6.54	-17.14	0.019
	Preoperative AST	25.92 ± 2.53		
	Postoperative AST	42.94 ± 5.27	-17.02	0.002
	Preoperative GGT	88.97 ± 20.61		
	Postoperative GGT	89.10 ± 15.64	-0.13	NS

N = Number; NS: Not significant; p: Probability; SE: Standard error.

Table 2 summarizes the differences between the changes in the preoperative and 1-day postoperative values for each test for both groups. There were no statistical differences for both groups in all LFTs. In addition, all patients had returned normal values of LFTs after 1 week postoperatively.

**Table 2.** The differences between Maryland and Hook dissectors in term of changes between one-day postoperative and preoperative liver function tests.

Test	Maryland dissector (mean for changes)	Hook dissector (mean for changes)	p-value
ALP	8.55	0.95	NS
Total bilirubin	-2.87	-3.25	NS
Direct bilirubin	-1.13	-1.14	NS
ALT	-17.14	-25.02	NS
AST	-17.02	-22.87	NS
GGT	-0.13	-6.04	NS

NS: Not significant; p: Probability.

## Discussion

To the best of our knowledge, this is the first study to evaluate the effect of different dissectors on alteration of LFTs after LC. As there were no statistical differences in the variation of LFTs between the Maryland and Hook, it seems that the dissector type has no effect on the alteration of LFTs.

The significant elevation of both ALT and AST points toward hepatocellular injury, which is contributed mostly to by the reduction in the hepatic blood flow due to an increase in intra-abdominal pressure [7–10]. There are two situations that can contribute to the alterations of LFTs: the increased intra-abdominal pressure and the use of CO2 to induce pneumoperitoneum. Pneumoperitoneum induces hepatic hypoperfusion by increasing intra-abdominal pressure. The normal portal blood pressure is approximately 8 mmHg, but the intra-abdominal pressure employed in laparoscopy can rise up to 16 mmHg. Pneumoperitoneum causes a reduction of portal venous inflow, marked reduction in liver perfusion and inadequate oxygen supply, with the consequence of hepatocellular injury [14]. This hepatocellular ischemic injury, due to increased abdominal pressure, can explain the elevation of ALT and AST as they are more sensitive and specific for hepatocellular injury [15]. Also, the anesthetic procedures and drugs used can induce hepatic toxic injury [16–18]. Moreover, CO2 employed to induce the pneumoperitoneum has a vasoconstrictive effect, which can reduce visceral blood flow [19]. Two studies by Hasukic *et al.* and Morino *et al.* demonstrated a pressure-dependent decrease in hepatic blood flow and enzyme elevations of ALT and AST [2,4]. However, postoperative bilirubin, ALP and GGT were not significantly elevated [9,20]. Eryilmaz *et al.* used indocyanine green elimination tests (ICG-PDR) as an indicator for liver function. In their trial, a significant decrease in ICG-PDR values in the standard pressure (14 mmHg) pneumoperitoneum was observed, when compared with the low-pressure group (10 mmHg) [21].

Furthermore, the alteration in LFTs could be contributed to by the use of electrocautery device. Hochstädetr *et al.* demonstrated a significant rise in liver enzymes (ALT and AST) after surgery, in both monopolar cutter and harmonic scalpel. However, postoperative values of these two enzymes were significantly higher in patients operated on using the monopolar cutter [22]. It is obvious that the usage of ultrasonic energy is better than electrocautery in LC in terms of shorter operating time, hospital stay sick leave and lower gallbladder perforation risk [23–26].

After these hypotheses and explanations, an important question arises: is it necessary to do routine testing of LFTs pre- and post-elective cases of LC? The assessment of preoperative LFTs is usually done to find if there is any hepatic disease or biliary obstructions in order to take the appropriate management. Tan *et al.* proposed that for patients with liver disease or any patients exhibiting altered LFTs before the surgery, laparoscopy might not be the right choice because it can further deteriorate hepatic function [3]. On the other hand, Pavlidis *et al.* suggested that LC can be safely carried out in patients with child classes A and B liver cirrhosis, without a significant increase in complications [27]. In fact, LC is not the only procedure associated with elevated postoperative LFTs. Other laparoscopic operations such as colorectal surgeries or other abdominal surgery have also been associated with altered postoperative LFTs [20,28]. Early elevation of LFTs after surgery usually returns to normal without intervention. Ahmad confirmed that mild-to-moderate elevation in preoperative LFTs may not be associated with any serious effect [29]. In the absence of clinical indications, routine preoperative or postoperative liver function testing is unnecessary [29]. In our study population, the LFTs for all patients returned to baseline level after 1 week.

Another factor that would play a role in the elevation of LFTs is the duration of surgery. Singal *et al.* demonstrated that the mean duration of surgery in the LC group was 57.7 min and in the open cholecystectomy group the mean duration of surgery was 61.8 min, which is nonsignificant (p = 0.109) and made both the groups comparable. In the LC group, they determined that the patient with minimum duration of surgery (40 min) had less elevation in liver enzymes (serum bilirubin, AST and ALT) as compared with the patient with maximum duration (90 min) of surgery [30].

## Conclusion

In LC, no differences were found between the utilization of Maryland and Hook dissectors in term of changes in LFTs. The elevation of LFTs seems to be attributable to other factors.

## Future perspective

As there is no difference between the Mayland and Hook dissectors, surgeons would have the choice to use any type of dissectors depending on their training and skills. Also, pneumoperitoneum would be the most important factor contributing to the elevation of LFTs after laparoscopic surgery. Further research should be conducted to find the optimal level of pressure. In addition, further investigations and studies should be performed to determine which patients should avoid laparoscopic surgery based on their perioperative clinical and laboratory status.

**Summary points**

- Elevation in levels of liver function tests following laparoscopic cholecystectomy is an apparent cause of apprehension to the surgeon.
- Pneumoperitoneum is a main contributing factor.
- No study was heretofore conducted to compare the effect of different techniques of dissection in laparoscopic cholecystectomy (Maryland vs Hook).
- This study includes patients who underwent laparoscopic cholecystectomy and were divided into two groups. Group A included patients who underwent dissection by Maryland dissecting forceps while group B underwent Hook dissecting instrument.
- Liver function tests were measured preoperatively and at 1 day and 1 week postoperatively.
- No statistical differences were seen in the variation of liver function tests between the Maryland and Hook.
- It seems that the dissector type has no effect on the alteration of liver function tests.

## References

[1] Cuschieri A Laparoscopic cholecystectomy. J. R. Coll. Surg. Edinb. 44(3), 187–190 (1999). 10372492

[2] Hasukic S, Kosuta D, Muminhodzic K Comparison of postoperative hepatic function between laparoscopic and open cholecystectomy. Med. Princ. Pract. 14(3), 147–150 (2005). 15863986 10.1159/000084630

[3] Tan M, Xu FF, Peng JS Changes in the level of serum liver enzymes after laparoscopic surgery. World J. Gastroenterol. 9(2), 364–367 (2003). 12532468 10.3748/wjg.v9.i2.364 PMC4611348

[4] Morino M, Giraudo G, Festa V Alterations in hepatic function during laparoscopic surgery: an experimental clinical study. Surg. Endosc. 12(7), 968–972 (1998). 9632872 10.1007/s004649900758

[5] Andrei VE, Schein M, Margolis M, Rucinski JC, Wise L Liver enzymes are commonly elevated following laparoscopic cholecystectomy: is elevated intra-abdominal pressure the cause? Dig. Surg. 15(3), 256–259 (1998). 9845595 10.1159/000018624

[6] Saber AA, Laraja RD, Nalbandian HI, Pablos-Mendez A, Hanna K Changes in liver function tests after laparoscopic cholecystectomy: not so rare, not always ominous. Am. Surg. 66(7), 699–702 (2000). 10917487

[7] Kotake Y, Takeda J, Matsumoto M, Tagawa M, Kikuchi H Subclinical hepatic dysfunction in laparoscopic cholecystectomy and laparoscopic colectomy. Br. J. Anaesth. 87(5), 774–777 (2001). 11878531 10.1093/bja/87.5.774

[8] Al-Jaberi TM, Tolba MF, Dwaba M, Hafiz M Liver function disturbances following laparoscopic cholecystectomy: incidence and significance. J. Laparoendosc. Adv. Surg. Tech. A 12(6), 407–410 (2002). 12590720 10.1089/109264202762252668

[9] Sakorafas G, Anagnostopoulos G, Stafyla V Elevation of serum liver enzymes after laparoscopic cholecystectomy. NZ Med. J. 118, 1317–1322 (2005). 15776093

[10] Omari A, Bani-Hani KE Effect of carbon dioxide pneumoperitoneum on liver function following laparoscopic cholecystectomy. J. Laparoendosc. Adv. Surg. Tech. A 17(4), 419–424 (2007). 17705719 10.1089/lap.2006.0160

[11] Halevy A, Gold-Deutch R, Negri M Are elevated liver enzymes and bilirubin levels significant after laparoscopic cholecystectomy in the absence of bile duct injury?. Ann. Surg. 219(4), 362–364 (1994). 8161261 10.1097/00000658-199404000-00006 PMC1243152

[12] Atila K, Terzi C, Ozkardesler S What is the role of the abdominal perfusion pressure for subclinical hepatic dysfunction in laparoscopic cholecystectomy?. J. Laparoendosc. Adv. Surg. Tech. A 19(1), 39–44 (2009). 19196087 10.1089/lap.2008.0085

[13] Clarke RS, Doggart JR, Lavery T Changes in liver function after different types of surgery. Br. J. Anaesth. 48(2), 119–128 (1976). 3192 10.1093/bja/48.2.119

[14] Lautt WW Mechanism and role of intrinsic regulation of hepatic arterial blood flow: hepatic arterial buffer response. Am. J. Physiol. 249(5 Pt 1), G549–G556 (1985). 3904482 10.1152/ajpgi.1985.249.5.G549

[15] Richter S, Olinger A, Hildebrandt U, Menger MD, Vollmar B Loss of physiologic hepatic blood flow control (“hepatic arterial buffer response”) during CO2 pneumoperitoneum in the rat. Anesth. Analg. 93(4), 872–877 (2001). 11574348 10.1097/00000539-200110000-00014

[16] Gopal DV, Rosen HR Abnormal findings on liver function tests. Interpreting results to narrow the diagnosis and establish a prognosis. Postgrad. Med. 107(2), 100–114 (2000). 10.3810/pgm.2000.02.869 10689411

[17] Augusti M, Elizalde I, Adalia R The effects of vasoactive drugs on hepatic blood fl ow changes induced by CO 2 laparoscopy: an animal study. Anesth. Analg. 93(5), 1121–1126 (2001). 11682379 10.1097/00000539-200111000-00011

[18] Cray SH, Crawford MW, Khayyam N, Carmichael FJL Effects of hypoxia and isofl urane on liver blood flow; the role of adenosine. Br. J. Anaesth. 86(3), 425–427 (2001). 11573535 10.1093/bja/86.3.425

[19] Jackimowicz J, Stultiens G, Smulders F Laparoscopic insufflation of the abdomen reduces portal venous flow. Surg. Endosc. 12(2), 129–133 (1998). 9479726 10.1007/s004649900612

[20] Nguyen NT, Braley S, Fleming NW, Lambourne L, Rivers R, Wolfe BM Comparison of postoperative hepatic function after laparoscopic versus open gastric bypass. Am. J. Surg. 186(1), 40–44 (2003). 12842747 10.1016/s0002-9610(03)00106-5

[21] Eryilmaz HB, Memiş D, Sezer A, Inal MT The effects of different insufflation pressures on liver functions assessed with LiMON on patients undergoing laparoscopic cholecystectomy. Sci. World J. 2012, 172575 (2012). 10.1100/2012/172575 PMC3349322 22619616

[22] Hochstädetr H, Bekavac-Beslin M, Doko M Functional liver damage during laparoscopic cholecystectomy as the sign of the late common bile duct stricture development. Hepatogastroenterology 50(51), 676–679 (2003). 12828058

[23] Wetter LA, Payne JH, Kirshenbaum G, Podoll EF, Bachinsky T, Way LW The ultrasonic dissector facilitates laparoscopic cholecystectomy. Arch. Surg. 127(10), 1195–1198 (1992). 1417485 10.1001/archsurg.1992.01420100053009

[24] Tsimoyiannis EC, Jabarin M, Glantzounis G, Lekkas ET, Siakas P, Stefanaki-Nikou S Laparoscopic cholecystectomy using ultrasonically activated coagulating shears. Surg. Laparosc. Endosc. 8(6), 421–424 (1998). 9864107

[25] Sietses C, Eljsbuts QAJ, von Blomberg BM, Ciesta MA Ultrasonic energy vs. monopolar electrocautery in laparoscopic cholecystectomy, influence on the postoperative systemic immune response. Surg. Endosc. 15(1), 69–71 (2001). 11178767 10.1007/s004640010061

[26] Janssen IMC, Swank DJ, Boonstra O, Knipscheer BC, Klinkenbijl JHG, Van Goor H Randomised clinical trials of ultrasonic versus electrocautery dissection of the gallbladder in laparoscopic cholecystectomy. Br. J. Surg. 90(7), 799–803 (2003). 12854103 10.1002/bjs.4128

[27] Pavlidis TE, Symeonidis NG, Psarras K Laparoscopic cholecystectomy in patients with cirrhosis of the liver and symptomatic cholelithiasis. JSLS 13(3), 342–345 (2009). 19793474 PMC3015965

[28] Jakimowicz J, Stultiens G, Smulders F Laparoscopic insufflation of the abdomen reduces portal venous flow. Surg. Endosc. 12(2), 129–132 (1998). 9479726 10.1007/s004649900612

[29] Ahmad NZ Routine testing of liver function before and after elective laparoscopic cholecystectomy: is it necessary? JSLS 15(1), 65–69 (2011). 21902946 10.4293/108680811X13022985131291 PMC3134700

[30] Singal R, Singal RP, Sandhu K Evaluation and comparison of postoperative levels of serum bilirubin, serum transaminases and alkaline phosphatase in laparoscopic cholecystectomy versus open cholecystectomy. J. Gastrointest. Oncol. 6(5), 479–486 (2015). 26487940 10.3978/j.issn.2078-6891.2015.058 PMC4570912

